# Dietary and physical activity recommendations to prevent type 2 diabetes in South Asian adults: A systematic review

**DOI:** 10.1371/journal.pone.0200681

**Published:** 2018-07-16

**Authors:** Mirthe Muilwijk, Mary Nicolaou, Samera A. Qureshi, Carlos Celis-Morales, Jason M. R. Gill, Aziz Sheikh, Naveed Sattar, Erik Beune, Anne Karen Jenum, Karien Stronks, Irene G. M. van Valkengoed

**Affiliations:** 1 Department of Public Health, Academic Medical Center, University of Amsterdam, Amsterdam, The Netherlands; 2 The Norwegian Centre for Migrant and Minority Health Research, Oslo, Norway; 3 Institute of Cardiovascular and Medical Sciences, University of Glasgow, Glasgow, United Kingdom; 4 Usher Institute of Population Health Sciences and Informatics, The University of Edinburgh, Edinburgh, United Kingdom; 5 Department of General Practice, Faculty of Medicine, Institute of Health and Society, Blindern, Oslo, Norway; Florida International University Herbert Wertheim College of Medicine, UNITED STATES

## Abstract

Intervention trials and guidelines for the prevention of type 2 diabetes (T2D) in populations of South Asian origin often include strategies to improve diet and physical activity that are based on those developed for other populations. These may be suboptimal for the South Asian target populations. We aimed to provide an overview of included recommended dietary and physical activity components, and to identify whether these were supported by evidence of their effectiveness. Databases were searched until September 2017 for intervention studies and guidelines with an adult South Asian population without T2D. The protocol was registered in PROSPERO, registration number: CRD42015207067. The quality of included studies and guidelines was assessed. Dietary and physical activity components, and effects on T2D incidence, glycemic status and adiposity measures, were summarized in tabular format and evaluated narratively. Eighteen intervention studies and four guidelines were identified. Dietary and physical activity components were similar to recommendations for the general population. Intervention studies and guidelines did not reference evidence to support the effectiveness of components included in the intervention for South Asian populations in particular. Moreover, we were unable to assess patterns of components to determine the effects of specific components. Evaluation of current and emerging components among South Asian populations and subgroups seems necessary to formulate more specific recommendations in future intervention studies and guidelines.

## Introduction

Populations of South Asian origin have a high risk for type 2 diabetes (T2D) and its complications, both in the country of origin and after migration [1–3]. In comparison with populations of European origin, South Asians have been shown to develop T2D at a younger age and at lower levels of adiposity [4, 5]. In the South Asian subcontinent, the diabetes epidemic has been fuelled by changes in the diet and patterns of physical activity due to economic transitions, especially in urban populations [6, 7]. As diet and physical activity are key modifiable risk factors for T2D [8], intervention studies [9–11] and guidelines [12] that aim to prevent or delay the onset of T2D include strategies to improve dietary intake and physical activity. For instance, the T2D prevention ‘Population and community-level interventions guideline’ issued by the National Institute for Health and Care Excellence (NICE) in the United Kingdom (UK), is aimed at dietary and physical activity components such as ‘adopt a low-fat diet’ and ‘use physically active forms of travel’ [12].

Recently, interventions targeting diet and physical activity have been developed to reduce the risk of T2D among populations of South Asian origin [13–17]. However, the trials evaluating these interventions only show small to moderate effects. We speculate that this may be related to the lack of targeting of recommended dietary and physical activity intervention components (hereinafter referred to as components) to the needs of South Asian populations. While many interventions reported cultural adaptation of the mode of delivery, and inclusion or exclusion of components based on the prevalence of certain health behaviours in the target population it is uncertain whether the included components were sufficiently targeted to specific characteristics of South Asian populations. In our own work, the components were often selected based on proven effectiveness of intervention studies conducted in other populations, mainly populations of European origin [13, 14]. Examples of intervention trials that followed this approach are Prevention of Diabetes & Obesity in South Asians (PODOSA) [17] and Diabetes prevention study in Hindustani Surinamese (DH!AAN) [13]. Using similar components may be appropriate in case these are also effective in South Asian populations, however it is also possible that the effects of specific changes in physical activity or diet is different across populations. There is some evidence to suggest that the effects of specific changes in physical activity or diet may be different in South Asian origin populations than in, for instance, those of European origin [18, 19]. For instance, a difference in the effect of a high-calorie, high-fat diet on insulin sensitivity was found between South Asian and White European origin men [18]. Other reports have suggested that the association between duration of physical activity and cardio-metabolic risk factors differs between South Asian and European origin men and women [19, 20]. While previous reviews have discussed the effects of interventions on the prevention of T2D, for instance by estimating the effects on weight loss, none have examined the specific components that were included in the interventions [8, 15, 21, 22].

Therefore, this systematic review set out to examine the dietary and physical activity components in intervention studies and in guidelines for the prevention of T2D in adult South Asian populations worldwide. Our broad aim was to identify gaps and, ultimately, stimulate the development of intervention studies and guidelines that are more targeted to the South Asian population. While public health messages are generally more consistent across populations, clinical guidelines may allow more specific recommendations for prevention at the individual level. The specific aims were to, first, describe the current dietary and physical activity components in intervention studies and guidelines. We included guidelines as these specify recommendations for South Asians based on graded evidence. Secondly, we determined whether the components are supported by population specific evidence. This evidence may include direct references to prior observational and experimental studies for each component listed in the intervention studies and guidelines. Additionally, intervention studies may provide insight in the effects of the included components.

## Materials and methods

The methods of the review were registered with the International Prospective Register of Systematic reviews (PROSPERO registration no CRD42015207067). A review protocol was published [23], and the methods are summarised here. The review was written in line with the Preferred Reporting Items for Systematic Reviews and Meta-Analyses (PRISMA) reporting guidelines (S1 PRISMA Checklist) [24].

We systematically searched PUBMED, Embase, Cochrane library and Web of Science from the start of the databases until September 12^th^, 2017. To the best of our knowledge no relevant studies were published before the start of these databases. All types of experimental, quasi-experimental and before-after studies and guidelines on dietary and/or physical activity components to prevent T2D in a population consisting of at least 75% South Asian adults (≥18 years) were considered. The search results were supplemented with reference list tracing of four key reviews [8, 15, 21, 22] and included studies [13, 16, 17, 25–39]. Study authors were contacted in case protocols or clarifications were desired, for instance to obtain the detailed recommended dietary and physical activity components. Corresponding authors of eleven of the studies were contacted to obtain more detailed information on the dietary components that were included in the intervention studies. Authors were additionally queried on which evidence components were based. Eight authors were reached, and provided additional information [13, 16, 17, 25–27, 33, 36]. Unpublished and in-progress studies were identified by searching English language trial registries. The applied search strategies are provided in the supplementary materials S1 Text. Additional studies were identified from experts in the field.

Guidelines were identified by searching online databases and searches of grey literature in all South Asian countries (India, Bangladesh, Pakistan, Sri Lanka, Nepal and Bhutan) and the top five countries to which South Asians have migrated with at least one million migrants a year (Saudi Arabia, United Kingdom, Kuwait, Oman, United States, United Arab Emirates, Canada and Qatar) [40]. The last search was conducted on November 8^th^, 2017. The applied search strategies are provided in S2 Text.

Two reviewers (MM, SAQ or IGMV) independently screened titles and abstracts for eligibility, based on the pre-set inclusion criteria. Thereafter full-texts were screened for eligibility by two reviewers (MM, SAQ or IGMV). For both processes a discussion was held in case of discrepancies and, if needed, a third reviewer was consulted to reach consensus (MN). A piloted data extraction form was developed in Microsoft Excel.

For each identified study, we reference the first identified manuscript reporting effects. However, information was extracted from all published manuscripts, study protocols and personal communication with authors. Only dietary and physical activity components that were explicitly mentioned in the written study documents or guidelines were extracted. Some studies referred to existing guidelines, for instance the National Institute of Nutrition (NIN) (India) guideline [41] and this reference was noted. However, components listed in referenced guidelines were not included in the data extraction. Data were extracted in duplicate by two reviewers (MM, SAQ, or IGMV) independently from each other. A discussion was held in case of discrepancies, and a third reviewer was consulted if needed (MN). In case data on dietary and / or physical activity strategy components was missing, published protocols were obtained and additional unpublished protocols were requested.

For intervention studies, the risk of bias was assessed at the study level using the Quality Assessment Tool for Quantitative Studies [42].The quality assessment of studies was done by three reviewers (MM, SAQ, CC or IGMV) instead of two reviewers as we perceived the quality assessment tool to be vulnerable to differences in interpretation. The general quality of guidelines was assessed by the Appraisal of Guidelines for Research & Evaluation II (AGREE II) instrument [43]. Assessment of the guidelines was done by two reviewers (MM, SAQ), subsections were rated between zero and seven which resulted in section scores between zero and 100, if needed a third reviewer (MN) was consulted. Diet and physical activity components were extracted from each identified study or guideline. Results were summarised in tabular form and evaluated narratively. The identified components were compared to the most recent NICE guideline for diabetes prevention [12] a general guideline listing the current components to prevent T2D in the U.K. to examine whether components in interventions and guidelines for South Asians were similar to those recommended for the general population or possibly targeted towards the South Asian population. The NICE guideline was selected for comparison as it is a recent and well regarded guideline for the general population in a European country with a large South Asian population. For each identified components, we examined whether the studies or guidelines supported the chosen component with specific evidence showing it to be relevant or effective for South Asians. Such evidence may include references to previous observational and experimental studies.

We explored whether certain components were recommended more frequently for pre-specified subgroups. Subgroups analysed were migrant / non-migrant groups (non-migrants were defined by populations living in South Asia), geographical setting (Europe / U.S. / Asia / Oceania) and ethnicity as described by the included study or guideline. We also considered differences by age and sex.

Patterns of effects of studies by their included intervention components were evaluated for studies with at least a moderate quality rating. We, first, described intervention effects on reported outcomes, and explored the consistency of effects. We then evaluated narratively whether differences existed between the effects of studies when certain components were included. Study outcomes were considered to be significantly improved if the p-value was <0.05. To achieve comparability across measures, the primary effect sizes of outcome measures per study were calculated by Cohen’s d, which was calculated by the mean difference or the difference in mean change divided by the pooled standard deviation. Cohen’s d values >0.8 were considered as large effect sizes, 0.5–0.79 as moderate and 0.2–0.49 as small [44]. Initially, comparisons of effects by components were only made if an outcome was reported by at least four studies that did and at least four studies that did not include a specific component. If a pattern was recognized, we also assessed the consistency across other outcomes of interest (separately listed in S1 Table). The cumulative evidence may be affected by publication bias, as it is conceivable that non-effective intervention studies were underreported. We checked the possibility of publication bias by listing all registered studies in the trial register. Bias was examined by distinguishing between studies that were yet ongoing and studies that had been completed for at least one year, but had not yet published results.

## Results

### Characteristics of identified studies

**Fig 1:**
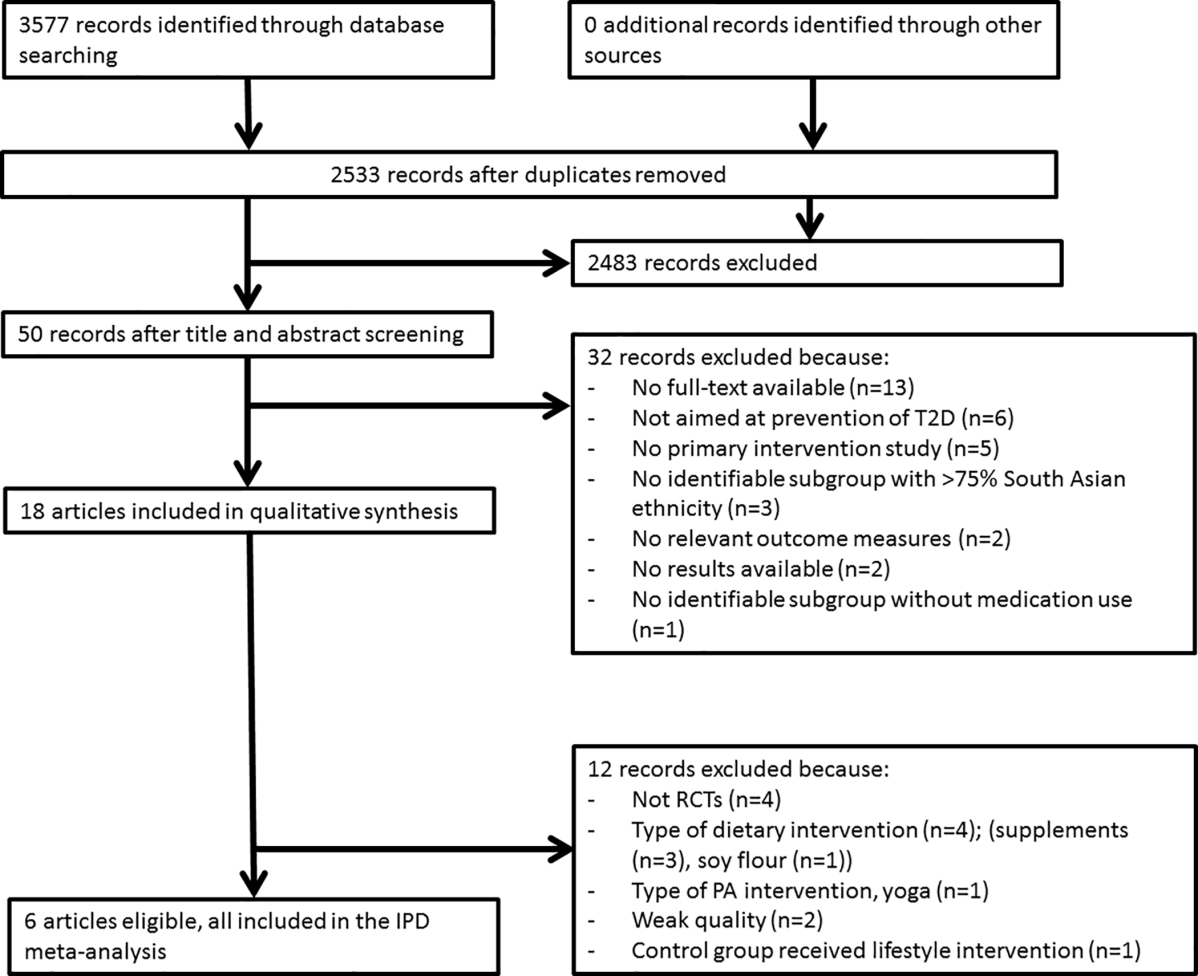
Study flow.

Our searches resulted in 3577 study records (Fig 1). No additional records were identified through other sources, although key reviews, reference lists of included papers and experts were consulted. After removing duplicates, 2533 records were left for title and abstract screening. From the 50 full-text records that were assessed for eligibility, 18 records were included in the review.

The majority (n = 14) of included studies were randomized controlled trials (RCTs; Table 1) with a study duration varying from 1.5 to 36 months [13, 16, 17, 28–33, 36–39]. Most studies (n = 11) were conducted in India [16, 25–29, 32, 35, 36, 38, 39]. In addition, four were conducted in Europe [13, 17, 31, 33], one in Bangladesh [37], one in New Zealand [30] and one in the U.S. [34] Most interventions combined dietary and physical activity components [13, 16, 17, 25–27, 32–34, 36, 38], but there were also interventions that focused solely on physical activity (n = 3) [29, 31, 35] or on diet alone (n = 4) [28, 30, 37, 39]. Most studies included South Asian populations of Indian ethnicity (n = 12) [16, 25–29, 31, 32, 35–38], had a wide age range (n = 11) [13, 17, 26–34, 37, 38], and focused specifically on those with pre-diabetes (n = 10) (impaired fasting glucose (IFG) or impaired glucose tolerance (IGT)) [13, 16, 17, 25, 28, 29, 32, 36, 37, 39].

**Table 1:** Study characteristics.

Study ID	Study design	Country	Ethnicity	Age (years)	% Male	Target group	Intervention size (n)	Study duration (months)	Behaviour change objective	Theory for behaviour change
**DH!AAN 2013 [13]**	RCT	Netherlands	South Asian Surinamese	18–60	51	IFG, IGT, glycated haemoglobin 42–46 mmol/l, HOMA-IR ≥2.39	536	12	Diet, PA	Theory of planned behaviour
**DPM 2008 [26]**	BAS	India	Indian	>10	41	All inhabitants	850	7	Diet, PA, Stress coping	Community based health promotion
**DPP 2012 [27]**	BAS	India	Indian	>18	46	All inhabitants	1681	6	Diet, PA, Stress coping	Community based health promotion
**Dutta et al. 2014 [28]**	RCT	India	Indian	30–80	41	IFG and/or IGT in 2 OGTTs	170	28	Diet	-
**Hegde et al. 2013 [29]**	RCT	India	Indian	30–75	48	Pre-diabetes, non-alcoholic, non-smoker	29	3	PA	Community based health promotion
**Hurst et al. 2010 [30]**	RCT	New Zealand	South Asian	23–68	0	Vitamin D deficiency	106	6	Diet	-
**IDPP-1 2006 [16]**	RCT	India	Indian	35–55	79	IGT on 2 OGTT	531	30	Diet, PA	Community based health promotion
**IDPP-2 2009 [36]**	RCT*	India	Indian	35–55	87	IGT on 2 OGTT	203	36	Diet, PA	-
**InnvaDiab-DE-PLAN 2012 [33]**	RCT	Norway	Pakistani	25–62	0	Not specified	198	7	Diet, PA	-
**Islam et al. 2016 [37]**	RCT	Bangladesh	Bangladeshi	30–65	51	Pre-diabetes	28	6	Diet	-
**Madsen et al. 2015 [35]**	LBW vs NBW	India	Indian	18–22	100	LBW	117	1.5	PA	-
**McDermott et al. 2014 [32]**	RCT	India	Indian	30–65	39	FBG ≥ 5.6 mmol/l 1^st^ degree relative T2D	41	2	Diet**, PA	-
**PAMH 2013 [31]**	RCT	Norway	Pakistani	25–60	100	Not physically active	150	5	PA	Social cognitive theory
**Patel et al. 2017 [38]**	RCT	US	Indian	>18	46	High risk T2D	36	6	Diet, PA	Community based health promotion
**PODOSA 2014 [17]**	RCT	UK	South Asian	35–80	46	WC ≥90 men ≥80 women, IGT or IFG	171	36	Diet, PA	Stages of change model
**Ramachandran et al. 2013 [25]**	RCT	India	Indian	35–55	100	IGT on 2 OGTT, mobile phone, able to read	537	20	Diet, PA	Stages of change
**RICE 2014 [34]**	Quasi-experimental	US	Indian	18–75	19	High risk T2D, adapted risk assessment tool ADA	126	6	Diet, PA, Stress coping	Community based health promotion
**Thirunavukkarasu et al. 2017 [39]**	RCT	India	Indian	40–60	0	Prediabetes, prehypertension	75	3	Diet	-
**AAPI Guide [46]**	Guideline	U.S.	South Asian	-		Not specified	-	-	Diet, PA	-
**Apnee Sehat [45]**	Guideline Community organization	UK	South Asian	-		Not specified	-	-	Diet, PA	-
**FHC [47]**	Guideline Health Centre	Canada	South Asian	-		Not specified	-	-	Diet, PA, Stress coping	-
**Misra et al. 2011 [48]**	Consensus guideline	India	South Asian	-		Not specified	-	-	Diet	-

* The control group in this study received lifestyle intervention and was therefore included in this systematic review.

**The focus of this study was a physical activity intervention, but dietary advice was provided as well.

### Characteristics of identified guidelines

No guidelines were identified by searching the predefined guideline databases, but four [45–47] were identified in the grey literature. In addition, one was excluded after initially being identified as a guideline, because it was a preliminary protocol for a planned intervention study [26]. Three of the identified guidelines targeted the general South Asian population in the UK or North America [45–47], the other guideline focused on India [48]. Three guidelines combined dietary and physical activity components [45–47], and one was solely focused on a dietary strategy [48] (Table 1).

### Risk of bias: quality assessment of the studies

In total, seven studies were rated strong [13, 16, 17, 25, 30, 31, 36], seven moderate [17, 26–29, 33, 34, 37] and four weak [32, 35, 38, 39] (Table 2). Overall moderate scores seemed mostly related to low ratings for blinding, and overall weak scores with low ratings for selection bias, design and confounders.

**Table 2:** Quality assessment intervention studies.

Study ID	Selection Bias	Design	Confounders	Blinding	Data Collection	Drop-Outs	Overall Rating
**DH!AAN [13]**	2	1	1	2	1	2	Strong
**DPM [26]**	1	2	1	3	1	2	Moderate
**DPP [27]**	1	2	1	3	1	1	Moderate
**Dutta et al. 2014 [28]**	1	1	1	3	1	1	Moderate
**Hegde et al. 2013 [29]**	1	1	1	3	1	1	Moderate
**Hurst et al. 2010 [30]**	1	1	1	1	1	2	Strong
**IDPP-1 [16]**	2	1	1	2	1	1	Strong
**IDPP-2 [36]**	2	1	1	1	1	1	Strong
**InnvaDiab-DE-PLAN [33]**	1	1	1	3	1	1	Moderate
**Islam et al. 2016 [37]**	3	1	1	1	1	1	Moderate
**Madsen et al. 2015 [35]**	3	3	3	2	1	1	Weak
**McDermott et al. 2014 [32]**	3	1	3	2	1	1	Weak
**PAMH [31]**	1	1	1	2	1	1	Strong
**Patel et al. 2017 [38]**	3	1	1	2	1	3	Weak
**PODOSA [17]**	1	1	1	2	1	1	Strong
**Ramachandran et al. 2013 [25]**	1	1	1	2	1	1	Strong
**RICE [34]**	2	2	2	3	1	1	Moderate
**Thirunavakkurasu et al. 2017 [39]**	3	1	3	2	1	3	Weak

1 = strong, 2 = moderate, 3 = weak. Studies without weak ratings are overall rated as strong, studies with one weak rating obtain an overall moderate rating and studies with two or more weak ratings are overall rated as weak.

### Risk of bias: General quality assessment of the guidelines

In general, guidelines scored high on stakeholder involvement, but low on rigour of development and editorial independence. Scores for the scope and purpose section varied from 44 to 100 points (Table 3). Examples of issues encountered in this section were a lack of attention to prevention of T2D (AAPI Guide [46]) and a lack of a clear description of the target group and no consideration of age groups (Apnee Sehat [45]). Further, rigour of development could be improved by the inclusion of references citing work that support the recommended components. Nevertheless, based on the overall assessment in the AGREE II tool, the guidelines could be qualified as acceptable for use.

**Table 3:** Quality assessment guidelines.

Guideline	Scope & Purpose	Stakeholder involvement	Rigour of development	Clarity of presentation	Applicability	Editorial independence
**AAPI Guide [46]**	67	94	25	94	38	50
**Apnee Sehat [45]**	44	100	8	72	88	25
**FHC [47]**	100	89	27	100	88	0
**Misra et al. 2011 [48]**	89	89	50	100	13	73

Guideline domains were rated on a scale from 0 to 100. According to the Agree II quality assessment tool, domain scores are independent and should therefore not be aggregated into a single quality score. Please note that an overall assessment is not given, as the overall guideline assessment does not include the domain scores.

### Dietary components included in intervention studies

In total, 15 of the 18 intervention studies used dietary components (Table 4). Of these, two studies focused solely on vitamin D supplementation [28, 30] one on zinc supplementation [37] and one on using soy flour [39]. The PODOSA study had a very detailed protocol and, therefore, listed the most dietary components (n = 25) [17]. We observed that most other studies listed some specific components in combination with a reference to existing general dietary guidelines or previous intervention studies [13, 16, 17, 25–27, 34, 36, 38].

**Table 4:** Dietary components in studies and guidelines on prevention of T2D in South Asian adults.

Studies^1^:	DH!AAN [13]^2^	DPM [26] DPP [27]^2^	Dutta et al. 2014 [28] Hurst et al. 2010 [30]	IDPP-1[16] Ramachandran et al. 2013 [25] IDPP-2 [36]^2^	InnvaDiab-DE-PLAN [33]	Islam et al. 2016 [37]	Patel et al. 2017 [38]	PODOSA [17]^2^	RICE [34]	Thirunavukkarasu et al. 2017 [39]	AAPI Guide [46]	Apnee Sehat [45]	FHC [47]	Misra et al. 2011 [48]
**Other trials or guidelines referred to**	[49–51]	[41]		[41]			[11, 52]	[11, 53, 54]	[11, 55, 56]					
**Any nutrients and foods components specified**	N,F	N,F	N,F	N,F	N,F	N	N, F	N,F	N,F	F	N,F	N,F	N,F	N,F
**Specific nutrients and foods:**														
**Protein**		N,F						N,F	N					N,F
**Fat**	N	F		N	N,F		N	N,F	F		N	N,F	N,F	N,F
**Carbohydrates**				N	N			N						N,F
**Sugar**		F		N	N,F			N,F	N,F		F	N	N,F	N,F
**Salt**				N	N			N	N,F			N	N,F	N,F
**Cholesterol**									N					N
**Vitamins and minerals**			N			N		N				N		N
**Fibre**	N	N		N				N			N		N	N
**Fruits**	F			F			F	F	F		F	F	F	F
**Vegetables**	F	F		F	F		F	F	F		F	F	F	F
**Whole grains**		F		F	F			F	F			F	F	F
**Cereal and grains**				F								F	F	F
**Legumes**		F		F	F			F		F		F		F
**Low GI foods**														F
**Alcohol**								F						F
**Nuts**								F				F	F	F
**Meat**								F					F	
**Fish**								F				F		F
**Dairy**								F					F	
**Dilute juices & choose fresh**												F		
**Cucumber instead of boondi raita**								F						
**Pickles and char alternatives**								F						
**Replace chevda with popcorn**								F						
**Any patterns specified**	P	P		P				P	P			P	P	P
**Specific patterns:**														
**Portion sizes**		P						P	P			P	P	P
**Eat breakfast**	P											P		P
**Cooking methods**												P		P
**Vegetarian diet**									P		P			
**Salt**									P					P
**Iron absorption**												P		
**Balanced meals**				P				P	P			P	P	
**Timing of meals**												P	P	P
**Limit eating out**									P				P	
**Energy deficit (500-600kcals/day)**								P						

N, F or P indicates that a component was formulated for the nutrient, food (a certain product) or pattern respectively. A more extended table is provided in the supplementary data (S4 Table).

^1^Studies in which the same components were recommended are shown in a combined column for reasons of space.

^2^Study leaders provided additional information on components.

The recommended dietary components in intervention studies were compared to the NICE guideline for T2D prevention, published May 2011 [12], as was explicated in the study protocol [23]. The NICE guideline mainly focused on starchy foods, fibre, low-fat diets and intake of fruits and vegetables, and further recommended to avoid fried products or products high in sugar, to minimise alcohol intake, to avoid increasing calorie intake, watch portion size and to use breakfast (S2 Table) [12].

The intervention studies were partly in line with the NICE guideline[12]. For instance, many specific components included in these studies were: fat or saturated fat (including fried products; n = 10) [13, 16, 17, 25–27, 33, 34, 36, 38], vegetables (n = 10) [13, 16, 17, 25–27, 33, 34, 36, 38], sugar (n = 8) [16, 17, 25–27, 33, 34, 36] whole grains (n = 8) [16, 17, 25–27, 33, 34, 36], fibre (n = 7) [13, 16, 17, 25–27, 36], legumes (n = 7) [16, 17, 25–27, 33, 36], fruits (n = 7) [13, 16, 17, 25, 34, 36, 38] and portion sizes (n = 4) [17, 26, 27, 34]. Only one study (DH!AAN [13]) recommended the consumption of breakfast, and one study recommended limiting alcohol and calorie intake (PODOSA [17]). Finally, some interventions also included advice on protein intake [17, 26, 27, 34, 38] (n = 5) and balanced meals [16, 17, 25, 34] (n = 4), while the NICE guideline did not [12]. We explored whether there were differences in patterns of recommended dietary components according to population subgroups over the various intervention studies, but did not find clear differences by migration status, ethnicity and geographical location (S1 Table). We were not able to explore differences in patterns according to sex and age due to a lack in variation of these variables across the studies.

### Dietary components included in guidelines

The identified South Asian guidelines also recommended components that were in line with the NICE guideline. However, the NICE guideline used more general descriptions, whereas the identified guidelines provided more detailed recommendations. Guidelines for instance recommend to “replace chevda with popcorn” while the NICE guideline recommends to “consume as little as possible fried food; drinks and confectionery high in added sugar; and other food high in fat and sugar”. In line with the NICE guideline, fat, sugar, fruits and vegetables were mentioned in all guidelines [45–48], while fibre [46–48], whole grains [45, 47, 48], cereal intake [45, 47, 48] and portion sizes [45, 47, 48] were listed in three out of four guidelines, only two out of four guidelines included legume intake [46, 48]. In contrast to the NICE guideline, three out of four identified guidelines included components on salt [45, 47, 48], nuts [45, 47, 48] and timing of meals [45, 47, 48].

### Physical activity components in intervention studies

Table 5 summarizes the physical activity components in the studies and guidelines. All studies, except the ones that focused on a single dietary component [28, 30, 37, 39], included physical activity (Table 5). The NICE guideline recommends to be physically active for at least 30 minutes per day on at least five days per week at least at a moderate-intensity (S2 Table) [12]. Most components in the intervention studies were in line with the recommendation to be active for at least 30 minutes per day for at least five days a week. However, there were three studies that recommended a longer duration of activity per day, varying from 45 to 90 minutes [29, 33, 35], and one recommended to be active on two days per week [33]. The preferred type of activity was specified in nine of the studies; walking was the most recommended activity [16, 17, 25, 33, 36]. Two studies were solely based on yoga [29, 32]. We explored whether there were differences in patterns of recommended physical activity components across subgroups (Table 5). Recommendations for duration, frequency and intensity did not systematically vary across studies among migrant versus non-migrant populations or other subgroups. The only observation that could be made was that studies conducted among non-migrants [16, 25–29, 36, 38] seemed to recommend specific activities more frequently, such as walking or yoga, than studies conducted among migrants.

**Table 5:** Physical activity components in studies and guidelines on prevention of T2D in South Asian adults.

Study ID	‘ / day	Days / week	Activity	Intensity
**DH!AAN [13]**	30	7		
**DPM [26]**	150 / week			Moderate
**DPP [27]**	30	7		
**Hegde et al. 2013 [29]**	75–90	5	Yoga	
**IDPP-1 [16]**	30	7	Brisk walking	Moderate
**IDPP-2 [36]**	30	7	Brisk walking	Moderate
**InnvaDiab-DE-PLAN [33]**	60	2	Walking 5000 steps	Low
**Madsen et al. 2015 [35]**	45	7	Cycling	
**McDermott et al. 2014 [32]**	32	3 to 6	Yoga	Moderate
**PAMH [31]**	30	7		Moderate
**Patel et al. 2017 [38]**	150 / week		Or walking 10.000 steps	Moderate
**PODOSA [17]**	30	7	Brisk walking	Moderate
**Ramachandran et al. 2013 [25]**	30	7	Brisk walking	Moderate
**RICE [34]**			Domestic activities	
**AAPI Guide [36]**	30	Most or all	Walking or other sports	Moderate
**Apnee Sehat [35]**	30	7	What makes happy	
**FHC [37]**	150 / week			Moderate

### Physical activity components in guidelines

Three out of four guidelines included physical activity (Table 5). A similar pattern of recommended physical activity duration and frequency as mentioned in the intervention studies was observed [45–47].

### Targeting of components to the characteristics of the South Asian population

We found that components used in studies were often based on prior T2D intervention studies in the general population, or on general population guidelines from the geographical area where the study was conducted (Tables 4 and 5). For instance, the dietary components that were recommended in the IDPP-1 study [16] were based on the National Institute of Nutrition (India) dietary guidelines, whereas the components in the DH!AAN study were based on the Dutch study on lifestyle intervention and impaired glucose tolerance Maastricht (SLIM) [51] and guidelines that were developed for the Dutch population [49, 50]. The majority of studies reported having adapted the recommendations to the cultural context of the population, in particular the mode of delivery of the intervention for instance by using South Asian dieticians [13, 17, 26, 27, 33, 34]. However, the studies did not state that components were selected or adapted based on specific evidence on the effects of components on T2D risk in the target population (e.g. people of Indian descent with IGT). We did not identify any referenced articles that state components to be effective within the South Asian population in either intervention studies or guidelines [13, 16, 17, 25–39, 45–48], nor did we identify evidence in original interventions or guidelines on which some intervention studies were based [11, 41, 49–51, 53–56].

### Patterns of effects of intervention studies

We included the 12 studies with a moderate to strong rating in the evaluation of the effects on T2D prevalence and its underlying measures (S1 Table), four studies were excluded due to a weak rating [32, 35, 38, 39]. The studies reported the effects with regard to a variety of different glucose metabolism and adiposity measures [13, 16, 17, 25–31, 33, 34]. Yet, only five studies reported effects on T2D incidence [16, 17, 25, 28, 36]. These were the studies with the longest study durations (20–36 months). In one study the intervention group received the lifestyle intervention combined with medication, while the control group received the lifestyle intervention [36], we considered the study arm that only received lifestyle intervention as a before-afer study. All studies reported lower progression rates to T2D in the intervention group versus the control group, although the difference in one study was not statistically significant [17] Results for fasting glucose and other measures of glucose metabolism were less consistent. We could include the effect size as determined by Cohen’s d for fasting glucose in nine studies; effect sizes varied from -0.14 to 2.78 standard deviations [13, 16, 17, 26–29, 33, 34]. We could not include the other three studies as standard deviations were not reported. All, but one study [30] reported on adiposity measures. We found effect sizes of -0.05 to 0.34 standard deviations [13, 16, 17, 26–29, 31, 33, 34] for BMI, which was included as an outcome measure in twelve studies of which for ten studies Cohen’s d could be calculated. Although not all results significantly improved, most results were in the direction of an improvement.

For some studies, strength of the effects were different for different types of outcomes [17, 31, 33, 34]. For example, PAMH [31] and PODOSA [17] found significant improved results on adiposity parameters, but not on glucose measures. In contrast, RICE [34] and InnvaDiab-DE-PLAN [33] found significant differences for glucose, but not for adiposity measures. However, the directionality of the effects was similar for the different outcomes.

The effects of studies according to the components protein, portion sizes, carbohydrates, fibre, fruits, legumes, portion sizes and balanced meals were evaluated for glucose, BMI and waist circumference measures (Table 6 and S3 Table). However, no clear conclusion could be drawn from these comparisons, as the number of studies was limited and components overlapped.

**Table 6:** Effects of components included in intervention studies.

Studies	Effects			Effects of components											
				Patterns / Portion sizes		Carbohydrates		Fibre		Fruits		Legumes		Balanced meals	
Glucose (mmol/l)	Base I	ES	p-value	Yes	No	Yes	No	Yes	No	Yes	No	Yes	No	Yes	No
DH!AAN [9]	5.3	-0.14	0.66		0 X		0 X	0 X		0 X			0 X		0 X
DPM [21]	5.2^2^	0.08	0.05	0 ✓			0 ✓	0 ✓			0 ✓	0 ✓			0 ✓
DPP [22]	5.3^2^	0.05	<0.001	0 ✓			0 ✓	0 ✓			0 ✓	0 ✓			0 ✓
Dutta et al. 2014 [23]	6.1^2^	0.41	0.02		1 ✓		1 ✓		1 ✓		1 ✓		1 ✓		1 ✓
Hegde et al. 2013 [24]	5.3	0.00	0.04		0 ✓		0 ✓		0 ✓		0 ✓		0 ✓		0 ✓
Hurst et al. 2010 [25]	4.7		0.82		X		X		X		X		X		X
IDPP-1 [12]	5.4	0.23	0.03		1 ✓	1 ✓		1 ✓		1 ✓		1 ✓		1 ✓	
IDPP-2 [36]	5.7	0.19	≥0.05		0 X	0 X		0 X		0 X		0 X		0 X	
InnvaDiab-DE-PLAN [30]	5.6	2.78	0.02		3 ✓	3✓			3 ✓		3 ✓	3 ✓			3 ✓
Islam et al. 2016 [37]	5.8	1.6	<0.001		3 ✓		3 ✓		3 ✓		3 ✓		3 ✓		3 ✓
PAMH [20]	5.3		0.30		X		X		X		X		X		X
PODOSA [13]	5.8	0.12	0.34	0 X		0 X		0 X		0 X		0 X		0 X	
RICE [26]	6.4^2^	0.70	<0.01	2 ✓		2 ✓			2 ✓	2 ✓			2 ✓	2 ✓	
**BMI (kg/m2)**	** **	** **	** **												
DH!AAN [9]	28.1	0.08	0.09		0 X		0 X	0 X		0 X			0 X		0 X
DPM [21]	20.6	-0.05	<0.01	0 X			0 X	0 X			0 X	0 X			0 X
DPP [22]	20.7	0.02	<0.01	0 ✓			0 ✓	0 ✓			0 ✓	0 ✓			0 ✓
Dutta et al. 2014 [23]	26.3	0.24	≥0.05		1 X		1 X		1 X		1 X		1 X		1 X
Hegde et al. 2013 [24]	27.2	0.13	0.01		0 ✓		0 ✓		0 ✓		0 ✓		0 ✓		0 ✓
IDPP-1 [12]	25.5	0.06	0.04		0 ✓	0 ✓		0 ✓		0 ✓		0 ✓		0 ✓	
IDPP-2 [36]	26.0	-0.14	≥0.05		0 X	0 X		0 X		0 X		0 X		0 X	
InnvaDiab-DE-PLAN [30]	29.4	0.34	0.18		1 X	1 X			1 X		1 X	1 X			1 X
PAMH [20]	27.1		<0.01		✓		✓		✓		✓		✓		✓
PODOSA [13]	30.6	0.11	0.01	0 ✓		0 ✓		0 ✓		0 ✓		0 ✓		0 ✓	
Ramachandran et al. 2013 [29]	25.8	0.00			0	0		0		0		0		0	
RICE [26]	27.8	0.20	0.08	0 X		0 X			0 X	0 X			0 X	0 X	
**WC (cm)**	** **	** **	** **												
DH!AAN [9]	94	0.09	0.50		0 X		0 X	0 X		0 X			0 X		0 X
DPM [21]	76^2^	0.43	0.001	1✓			1 ✓	1 ✓			1 ✓	1 ✓			1 ✓
DPP [22]	75^2^	0.05	0.01	0 ✓			0 ✓	0 ✓			0 ✓	0 ✓			0 ✓
Hegde et al. 2013 [24]	88.9	-0.02	0.03		0 ✓		0 ✓		0 ✓		0 ✓		0 ✓		0 ✓
IDPP-1 [12]	89.1	0.07	0.43		0 X	0 X		0 X		0 X		0 X		0 X	
IDPP-2 [36]	91.2	0.14	≥0.05		0 X	0 X		0 X		0 X		0 X		0 X	
InnvaDiab-DE-PLAN [30]	95.3	0.62	0.18		1 X	1 X			1 X		1 X	1 X			1 X
PAMH [20]	98		0.01		✓		✓		✓		✓		✓		✓
PODOSA [13]	102.7	0.16	0.01	0 ✓		0 ✓		0 ✓		0 ✓		0 ✓		0 ✓	
Ramachandran et al. 2013 [29]	92.6	-0.01			0	0		0		0		0		0	
RICE [26]	93^2^	0.19	0.39	0 X		0 X			0 X	0 X			0 X	0 X	

1, 2 or 3 are shown to indicate the magnitude of the effect size, which corresponds to none (<0.2), small (>0.2), medium (>0.5) or large (0.8) A ✓ is shown to indicate that the study reported a significant improved p-value and a X is shown to indicate that there was no significant improved p-value. Yes and no were used to indicate whether the component was included in the study. Studies on vitamin D and physical activity were included in the evaluation of effects of components but did not include any of the evaluated components. T2D, type 2 diabetes; 2-h glucose, 2-h post 75g glucose blood glucose; IGT, Impaired Glucose Tolerance; IFG, Impaired Fasting Glucose; BMI, Body Mass Index; WC, Waist Circumference; HC, Hip Circumference; TC, Thigh Circumference; WHR, Waist Hip Ratio; BF, Body Fat; Out, Outcome; I, Intervention group; C, Control group; Base, Baseline; ES, Effect size shown by Cohens d.

^2^Original units were converted to SI units.

### Risk of publication bias

We identified 13 additional studies from trial registers that had not been published (Table 7) [57]. Based on the reported end date, two studies are still ongoing. Three studies ended recently (in 2016 or 2017). The remaining eight studies have not made their results public, although the studies had an estimated end date between 2009 and 2015. This suggests the possibility of a publication bias. Although the reason for this underreporting is not clear, it may be related to unsuccessful inclusion, discontinuation or failure to publish negative results.

**Table 7:** Overview additional studies.

Study ID	Ref	End date	Country	Ethnicity	Group	Age (years)	Behaviour change objective	Study type	Study duration (months)
**Badaam et al.**	[57]	03/2013	India	Indian	Pre-diabetes	25–40	PA; Aerobic vs. Sprint	RCT	3
**BanglaDiP**	[58]	10/2012	UK	Bangladeshi	At risk T2D	20–70	Diet, PA, Tobacco	RCT	6
**DIAbetes PREvention programme**	[59]	12/2012	India	Indian	IFG/IGT	18–80	Diet, PA (Yoga)		12
**GO for IT**	[60]	02/2016	UK	South Asian	At risk T2D	50–75	PA (HIIT)	Crossover trial	7 hours
**IPDS**	[61]	02/2017	India	Indian	BMI ≥23; IFG/IGT	30–70	PA (Fenugreek/Yoga)	RCT	36
**K-DPP**	[62, 63]	11/2015	India	Indian		30–60		RCT	24
**mDiab**	[64]	Ongoing	India	Indian	BMI ≥23	20–65	Diet, PA	RCT	3
**PREVENT-WIN**	[65]	12/2015	India	Indian	Pre-diabetes	20–60	Diet (Vitamin D)	RCT	24
**Ranasinghe et al. 2012**	[66, 67]	06/2012	Sri Lanka	Sri Lankan	Pre-diabetes	12–65	Diet (Zinc)	RCT	2
**Shekar et al.**	[68]	12/2015	India	Indian	IGT; Sedentary lifestyle	24–50	Lifestyle	RCT	2
**Thomson et al.**	[69]	12/2017	India/ UK		Pre-diabetes	18–74		RCT	24
**Velho et al.**	[70]	06/2016	India	Indian	Pre-diabetes	30–45	Diet	RCT	6
**VITALITY**	[71]	10/2013	UK	South Asian	Insulin resistance	25–75	Diet (Vitamin D)	RCT	6

IFG, Impaired fasting glucose; T2D, Type 2 diabetes; BMI, Body Mass Index; IGT, Impaired fasting glucose; PA, Physical activity; HIIT, High Intensity Interval Training; RCT, Randomised Controlled Trial.

## Discussion

This review found that most intervention studies and guidelines to prevent T2D in South Asians recommended a wide variety of dietary and physical activity components that were underpinned by evidence from studies in non-South Asian populations and that were largely in line with guidelines not specifically developed for a South Asian origin population, such as the NICE guideline. We could not identify clear patterns in components across subgroups according to study or population characteristics. The included studies and guidelines often did not provide specific evidence that recommended components were effective in reducing T2D risk among South Asian populations or subgroups. Although the overall directionality of the results was towards an improvement of outcome measures, we were not able to assess patterns to determine the contribution of specific components to these effects.

As anticipated, we found a lack of specific adaptations of components and a lack of underpinning of the suitability of the components for the target group in both intervention studies and guidelines. For some components this may be because evidence of potential effectiveness within this population was lacking at the time of development, but the lack of evidence for the effectiveness of components among South Asians was not mentioned in the studies or guidelines. Nor did these studies, in absence of specific data on effectiveness, reference specific observational data supporting the importance of the components among South Asians. However, evidence that was available may also not have been considered because researchers were optimistic concerning the expected effects based on the large reductions in T2D risk observed in efficacy studies [10, 11], including one from India [16]. The assumption that expected effects may be similar across populations may hold for several components, as confirmed by some observational work in South Asian populations. For example, a dietary pattern high in fruits, vegetables, dairy products and monounsaturated fatty acids and low in refined cereals was associated with a lower risk for T2D [72]. Another study showed reduced T2D risk for legume intake [73]. However, evidence for the effectiveness of other specific components is, as yet, unavailable. Moreover, the specific dose or change that is needed to reduce the risk for T2D in South Asian populations may differ from those in the European population. For instance, the optimal required amount and type of physical activity that is necessary to achieve an effect may differ by ethnic group [19]. We recommend further evaluations of the effectiveness of components within South Asian populations so that, if necessary, targeted recommendations can be developed for these populations. The recommendations should then also take into account the existing dietary patterns and activity levels in South Asian populations. South Asians, for instance, tend to be less physically active, and to more often have a vegetarian diet than populations of European origin [74–76]. In addition, dietary patterns may vary across by South Asian population (e.g. by country of residence) [77].

It is noteworthy that potentially relevant evidence that has only emerged in recent years was lacking from the intervention studies and guidelines for South Asians. These components may also be relevant to the general population, but were shown effective for South Asians. One example related to diet is the infrequent consideration of lacto-, lacto-ovo and semi-vegetarian diets [78]. A study conducted among men and women in India indicated that these types of diets were protective for T2D compared to non-vegetarian diets. An example related to physical activity is the lack of attention to sedentary time, although this might also be relevant for other populations. Emerging evidence among Pakistani immigrant men living in Norway indicates that a reduction of sedentary time may be as important as, or perhaps even more important than, an increase in moderate physical activity to reduce the T2D risk [79]. Secondary analyses of PODOSA reported a cross-sectional relationship between sitting time and 2-h glucose levels in U.K. South Asians [80]. These recent findings may be included in future intervention studies and guidelines, and it might be expected that their inclusion may increase effectiveness. While sedentary behaviour has recently been specified in the health guidelines of many countries they have yet to be included in guidelines / recommendations targeting South Asians.

Information on the extent to which recommendations were truly adopted in daily practice was not available. A change in T2D risk, given theoretical effects of the component, can only be observed when the behaviour of participants is changed towards recommended levels. Ram et al., 2014 showed that among those with greater compliance to the lifestyle modification than among participants that failed to make any changes, incidence of T2D was lower [81]. Unfortunately, such information on implementation and changes in behaviour were not reported for most studies limiting our ability to assess this further.

A general point that follows from our work is that given the high burden of T2D and T2D related complications among South Asians, guidelines to reduce the risk of T2D seem imperative. Yet, only four guidelines aimed at T2D prevention among South Asians were retrieved. This lack of guidelines aiming to reduce the risk of T2D among the high risk South Asian population fits the more general finding that there is a lack of consideration of ethnicity in reviews and health guidelines [82]. On the other hand, dissemination of guidelines for different types of subgroups may be difficult as different health messages for subgroups may lead to confusion across populations. However, identified guidelines were especially develop for South Asians and still did not recommended components especially developed for South Asians. Guidelines were not underpinned by evidence that showed components to be effective for South Asians. At the same time, the identified guidelines may not reflect all recommended components within the South Asian population as we may have missed recommendations that were only spread through other methods of dissemination. For instance, during the searches of the grey literature we identified that strategies and components to prevent T2D in South Asian countries are often communicated via alternative materials such as posters [83–85]. Also, more general (not specifically focused at the prevention of T2D) dietary and physical activity guidelines are available such as the Indian dietary guidelines, and the guideline for physical activity in Asian Indians [41], [86].

### Limitations

Our work has some limitations that merit discussion. Firstly, the search strategy for scientific studies was limited to abstracts written in English. Although we did the utmost to identify further work through reference tracing and expert consultations, this means that we may have missed studies that were not published or referenced in the English language. Similarly, we may also have missed some guidelines as, even though language was not a formal requirement, the search terms were in English.

Secondly, within studies some of the dietary and physical activity components may have been missed which should be considered in the interpretations of the findings. Although study investigators were contacted to retrieve protocols, not all were reached or investigators no longer had protocols available for assessment. In addition, we did not extract components listed in referenced material (e.g. general guidelines reference in the study) as referenced materials may have been used as basis for the trial design, but not fully included in the trial.

Thirdly, we used the Quality Assessment Tool for Quantitative Studies [42] to assess study quality and only included studies in the evaluation of effectiveness if the study obtained at least a moderate rating. However, studies of poor quality were not excluded from the assessment of diet and physical activity components. Although the execution of the study does not directly reflect the intervention development process, it is not unlikely that the design of an intervention from a study with a poor rating is also of poorer quality than that of other studies. Another point is that we observed the Quality Assessment Tool to be vulnerable to minor differences in interpretation, while relatively arbitrary changes in rating of one section can result in a different overall score. Previously, the inter-rater agreement for individual domains was described as fair [87]. Due to the discrepancies in the scoring, it was decided to involve a third reviewer in the scoring of all studies. This also better addressed the point that reviewers were not blinded to the studies. Last, based on the estimated end date of yet unpublished trials, we found evidence to suggest possible publication bias. We found six studies that ended prior to 2014 that have not yet updated their registrations or published their results in the international scientific literature. Although the reason for this underreporting cannot be determined, e.g. unsuccessful inclusion or negative results, it is indicative of a probable publication bias.

## Conclusions

In conclusion, our study shows that, as was expected, dietary and physical activity components in intervention studies and guidelines to prevent T2D in South Asians are based upon those developed for the European white population. Intervention studies and guidelines did not reference evidence that shows included components to be effective for South Asian populations in particular. It remains uncertain whether dietary and/or physical activity components in interventions and guidelines should be adapted to fit the metabolic characteristics of South Asians, or whether other aspects must be adapted to increase the effectiveness of interventions. Therefore, we cannot yet formulate recommendations to include or exclude certain components in future intervention studies. Research into the effects of current and emerging components among South Asian populations and subgroups in their specific contexts is needed for further clarification, or for instance by more high quality trials in South Asians that test a specific dietary or physical activity component.

## Supporting information

S1 PRISMA Checklist (DOC)

S1 Text Search strategy scientific literature databases. (DOC)

S2 Text Search strategy guideline databases. (DOC)

S1 Table Effects of studies. ^1^ 0, 1, 2 or 3 are shown to indicate the magnitude of the effect size, which corresponds to none (<0.2), small (>0.2), medium (>0.5) or large (0.8) A √ is shown to indicate that the study reported a significant improved p-value and a X is shown to indicate that there was no significant improved p-value. Yes and no were used to indicate whether the component was included in the study. Studies on vitamin D and physical activity were included in the evaluation of effects of components but did not include any of the evaluated components. T2D, type 2 diabetes; 2-h glucose, 2-h post 75g glucose blood glucose; IGT, Impaired Glucose Tolerance; IFG, Impaired Fasting Glucose; BMI, Body Mass Index; WC, Waist Circumference; HC, Hip Circumference; TC, Thigh Circumference; WHR, Waist Hip Ratio; BF, Body Fat; Out, Outcome; I, Intervention group; C, Control group; Base, Baseline; ES, Effect size shown by Cohens d *Original units were converted to SI units. Studies shown in cursive obtained a weak score in the quality assessment and were not included in the effects assessment.
(DOC)

S2 Table Recommended components NICE guideline. (DOC)

S3 Table Patterns according to included components. Data are the number of studies reporting the specific component. Components were included in the table, if these were recommended by more than one study.
(DOC)

S4 Table Dietary components per study compared to the NICE guideline. (DOC)
