# Supplementary material for: Dietary and physical activity recommendations to prevent type 2 diabetes in South Asian adults: A systematic review
Source: PLoS One. 2018 Jul 16;13(7):e0200681. doi: 10.1371/journal.pone.0200681 (PMC6047810; doi:10.1371/journal.pone.0200681)
Supplement: S1 Text — (DOC) [file pone.0200681.s002.doc]

**S1 File. Search strategy scientific literature databases.**

Search strategy PUBMED:

(("Asia, Western"[Mesh:NoExp] OR "Bangladesh"[Mesh] OR "Sri Lanka"[Mesh] OR "Nepal"[Mesh] OR "Bhutan"[Mesh] OR "India"[Mesh] OR "Pakistan"[Mesh] OR South Asia*[tiab] OR Asian India*[tiab] OR Pakistan*[tiab] OR Bangladesh*[tiab] OR Sri Lanka*[tiab] OR Nepal*[tiab] OR Bhutan*[tiab] OR India*[tiab]) AND ("Diabetes Mellitus"[Mesh:NoExp] OR "Diabetes Mellitus, Type 2"[Mesh] OR "Overweight"[Mesh] OR "Obesity"[Mesh] OR diabet*[tiab] OR obes*[tiab] OR overweight[tiab] OR weight[tiab]) AND ("Diet"[Mesh] OR "Diet Therapy"[Mesh] OR "Eating"[Mesh] OR "Feeding Behavior"[Mesh:NoExp] OR "Food Habits"[Mesh] OR "diet therapy" [Subheading] OR intake[tiab] OR food*[tiab] OR diet*[tiab] OR nutrition*[tiab] OR eat*[tiab] OR "Exercise"[Mesh] OR "Exercise Therapy"[Mesh] OR "Physical Fitness"[Mesh] OR "Sports"[Mesh] OR activ*[tiab] OR sport*[tiab] OR exercis*[tiab] OR walk*[tiab] OR lifestyle modification[ti]) AND ("Primary Prevention"[Mesh] OR "prevention and control" [Subheading] OR "Guideline" [Publication Type] OR guideline* OR intervention*[tiab] OR advice*[tiab] OR recommendation*[tiab] OR prevent*[tiab] OR promot*[tiab] OR support*[tiab]) AND ("Clinical Trial" [Publication Type] OR "Clinical Trials as Topic"[Mesh] OR "Cohort Studies"[Mesh] OR "Case-Control Studies"[Mesh] OR "Intervention Studies"[Mesh] OR "Feasibility Studies"[Mesh] OR "Case Reports" [Publication Type] OR "Clinical Conference" [Publication Type] OR "Comparative Study" [Publication Type] OR "Consensus Development Conference" [Publication Type] OR "Evaluation Studies" [Publication Type] OR "Meta-Analysis" [Publication Type] OR "Multicenter Study" [Publication Type] OR "Validation Studies" [Publication Type] OR "Observational Study" [Publication Type] OR "Pilot Projects"[Mesh] OR "Controlled Before-After Studies"[Mesh] OR before after stud*[tiab] OR trial[ti] OR controlled[ti] OR random*[tiab] OR intervention*[tiab] OR cohort*[tiab] OR compar*[tiab] OR control*[tiab] OR experimental stud*[tiab])) NOT ("Child"[Mesh] NOT "Adult"[Mesh]) NOT (("Animals"[Mesh] NOT "Humans"[Mesh]) OR "Animals, Laboratory"[Mesh] OR "Animal Experimentation"[Mesh] OR "Models, Animal"[Mesh] OR "Rodentia"[Mesh] OR rat[ti] OR rats[ti] OR mouse[ti] OR mice[ti] OR "Editorial" [Publication Type] OR "Letter" [Publication Type] OR "News" [Publication Type] OR "Comment" [Publication Type] OR "Historical Article" [Publication Type] OR "Anecdotes as Topic"[Mesh] OR letter*[ti] OR comment*[ti] OR abstracts[ti])

Search strategy Embase (Ovid):

| **#** | Searches |
| --- | --- |
| 1 | south asia/ or bangladesh/ or bhutan/ or india/ or nepal/ or pakistan/ or sri lanka/ or (South Asia* or Asian India* or Pakistan* or Bangladesh* or Sri Lanka* or Nepal* or Bhutan* or India*).ti,ab,kw. |
| 2 | *diabetes mellitus/ or *non insulin dependent diabetes mellitus/ or exp *obesity/ or (diabet* or obes* or overweight or weight).ti,ab,kw. |
| 3 | exp *diet/ or exp *diet therapy/ or *food intake/ or *eating/ or exp *dietary intake/ or exp feeding behavior/ or (intake or food* or diet* or nutrition* or eat*).ti,ab,kw. or exp exercise/ or exp *kinesiotherapy/ or *fitness/ or exp *sport/ or physical activity/ or (activ* or sport* or exercis* or walk*).ti,ab,kw. or lifestyle modification.ti. |
| 4 | *prevention/ or "prevention and control"/ or primary prevention/ or pc.fs. or exp practice guideline/ or (guideline* or intervention* or advice* or recommendation* or prevent* or promot* or support*).ti,ab,kw. |
| 5 | exp clinical trial/ or exp controlled clinical trial/ or randomized controlled trial/ or clinical study/ or "clinical trial (topic)"/ or exp "randomized controlled trial (topic)"/ or case study/ or clinical study/ or cohort analysis/ or retrospective study/ or prospective study/ or exp comparative study/ or exp controlled study/ or experimental study/ or observational study/ or prevention study/ or validation study/ or "systematic review"/ or multicenter study/ or pilot study/ or (before adj10 (after or during)).ti,ab. or (trial or controlled).ti. or random*.ti,ab. or ((experiment* or quasi* or random* or control) adj3 (method* or study or trial or design*)).ti,ab,kw. |
| 6 | child/ not adult/ |
| 7 | (animal/ not human/) or (exp experimental animal/ or animal experiment/ or animal model/ or exp rodent/ or editorial/ or letter/ or literature/ or (letter* or comment* or abstracts).ti.) |
| 8 | (1 and 2 and 3 and 4 and 5) not 6 not 7 |

Search strategy Cochrane library:

ID Search

#1 MeSH descriptor: [Asia, Western] this term only

#2 MeSH descriptor: [Bangladesh] explode all trees

#3 MeSH descriptor: [Sri Lanka] explode all trees

#4 MeSH descriptor: [Nepal] explode all trees

#5 MeSH descriptor: [Bhutan] explode all trees

#6 MeSH descriptor: [India] explode all trees

#7 MeSH descriptor: [Pakistan] explode all trees

#8 South Asia* or Asian India* or Pakistan* or Bangladesh* or Sri Lanka* or Nepal* or Bhutan* or India*:ti,ab,kw (Word variations have been searched)

#9 MeSH descriptor: [Asia, Western] explode all trees

#10 #1 or #2 or #3 or #4 or #5 or #6 or #7 or #8

#11 MeSH descriptor: [Diabetes Mellitus] this term only

#12 MeSH descriptor: [Diabetes Mellitus, Type 2] explode all trees

#13 MeSH descriptor: [Overweight] explode all trees

#14 diabet* or obes* or overweigh*:ti,ab,kw (Word variations have been searched)

#15 #11 or #12 or #13 or #14

#16 MeSH descriptor: [Diet] explode all trees

#17 MeSH descriptor: [Diet Therapy] explode all trees

#18 MeSH descriptor: [Eating] explode all trees

#19 MeSH descriptor: [Feeding Behavior] explode all trees

#20 intake or food* or diet* or nutrition* or eat*:ti,ab,kw (Word variations have been searched)

#21 MeSH descriptor: [Exercise] explode all trees

#22 MeSH descriptor: [Exercise Therapy] explode all trees

#23 MeSH descriptor: [Physical Fitness] explode all trees

#24 MeSH descriptor: [Sports] explode all trees

#25 MeSH descriptor: [Motor Activity] explode all trees

#26 MeSH descriptor: [Physical Education and Training] explode all trees

#27 activ* or sport* or exercis* or walk*:ti,ab,kw (Word variations have been searched)

#28 #16 or #17 or #18 or #19 or #20 or #21 or #22 or #23 or #24 or #25 or #26 or #27

#29 MeSH descriptor: [Primary Prevention] explode all trees

#30 MeSH descriptor: [Guideline] explode all trees

#31 guideline* or intervention* or advice* or recommendation* or prevent* or promot* or support*:ti,ab,kw (Word variations have been searched)

#32 MeSH descriptor: [Health Planning Guidelines] explode all trees

#33 #29 or #30 or #31 or #32

#34 #10 and #15 and #28 and #33

Search strategy Web of Science:

| # 5 | #4 AND #3 AND #2 AND #1  Indexes=SCI-EXPANDED, SSCI Timespan=All years |
| --- | --- |
| # 4 | TITLE: (diabet* OR obes* OR overweight OR weight)  Indexes=SCI-EXPANDED, SSCI Timespan=All years |
| # 3 | TOPIC: (guideline* OR intervention* OR advice* OR recommendation* OR prevent* OR promot* OR support*)  Indexes=SCI-EXPANDED, SSCI Timespan=All years |
| # 2 | TOPIC: (intake OR food* OR diet* OR nutrition* OR eat* OR activ* OR sport* OR exercis* OR walk*)  Indexes=SCI-EXPANDED, SSCI Timespan=All years |
| # 1 | TITLE: (South Asia* OR Asian India* OR Pakistan* OR Bangladesh* OR Sri Lanka* OR Nepal* OR Bhutan* OR India*)  Indexes=SCI-EXPANDED, SSCI Timespan=All years |
