# Supplementary material for: Dietary and physical activity recommendations to prevent type 2 diabetes in South Asian adults: A systematic review
Source: PLoS One. 2018 Jul 16;13(7):e0200681. doi: 10.1371/journal.pone.0200681 (PMC6047810; doi:10.1371/journal.pone.0200681)
Supplement: S2 Text — (DOC) [file pone.0200681.s003.doc]

**S2 Text. Search strategy guideline databases.**

Search strategy free-field format:

(South Asia* OR Asian India* OR Pakistan* OR Bangladesh* OR Sri Lanka* OR Nepal* OR Bhutan* OR India*)

AND

(diabet* OR obes* OR overweight OR weight)

AND

(intake OR food* OR diet* OR nutrition* OR eat* OR activ* OR sport* OR exercis* OR walk*)
