# Supplementary material for: Dietary and physical activity recommendations to prevent type 2 diabetes in South Asian adults: A systematic review
Source: PLoS One. 2018 Jul 16;13(7):e0200681. doi: 10.1371/journal.pone.0200681 (PMC6047810; doi:10.1371/journal.pone.0200681)
Supplement: S1 Table — 1 0, 1, 2 or 3 are shown to indicate the magnitude of the effect size, which corresponds to none (<0.2), small (>0.2), medium (>0.5) or large (0.8) A √ is shown to indicate that the study reported a significant improved p-value and a X is shown to indicate that there was no significant improved p-value. Yes and no were used to indicate whether the component was included in the study. Studies on vitamin D and physical activity were included in the evaluation of effects of components but did not include any of the evaluated components. T2D, type 2 diabetes; 2-h glucose, 2-h post 75g glucose blood glucose; IGT, Impaired Glucose Tolerance; IFG, Impaired Fasting Glucose; BMI, Body Mass Index; WC, Waist Circumference; HC, Hip Circumference; TC, Thigh Circumference; WHR, Waist Hip Ratio; BF, Body Fat; Out, Outcome; I, Intervention group; C, Control group; Base, Baseline; ES, Effect size shown by Cohens d *Original units were converted to SI units. Studies shown in cursive obtained a weak score in the quality assessment and were not included in the effects assessment. (DOC) [file pone.0200681.s004.doc]

**S1 Table. Effects of studies.**

| **Studies** | **Effects studies** | | | | | | **Effects components** | | | | | | | | | | | |
| --- | --- | --- | --- | --- | --- | --- | --- | --- | --- | --- | --- | --- | --- | --- | --- | --- | --- | --- |
|  |  |  |  |  |  |  | **Patterns/**  **Portion sizes** | | **Carbohydrates** | | **Fibre** | | **Fruits** | | **Legumes** | | **Balanced meals** | |
| **% T2D** | **Base C** | **Out C** | **Base I** | **Out I** | **ES** | **p-value** | **Yes** | **No** | **Yes** | **No** | **Yes** | **No** | **Yes** | **No** | **Yes** | **No** | **Yes** | **No** |
| Dutta et al. 2014[28] |  | 27 |  | 10 |  | 0.04 |  | 🗸 |  | 🗸 |  | 🗸 |  | 🗸 |  | 🗸 |  | 🗸 |
| IDPP-1[16] |  | 55 |  | 39 |  | 0.02 | 🗸 |  | 🗸 |  | 🗸 |  | 🗸 |  |  | 🗸 | 🗸 |  |
| IDPP-2 [36] |  |  |  | 32 |  |  |  |  |  |  |  |  |  |  |  |  |  |  |
| PODOSA[17] |  | 21 |  | 15 |  | 0.37 |  | X | X |  |  | X | X |  |  | X | X |  |
| Ramachandran et al. 2013[25] |  | 27 |  | 18 |  | 0.02 |  | 🗸 | 🗸 |  | 🗸 |  | 🗸 |  | 🗸 |  | 🗸 |  |
| **Glucose (mmol/l)** |  |  |  |  |  |  |  |  |  |  |  |  |  |  |  |  |  |  |
| DH!AAN[13] | 5.3 | 4.8 | 5.3 | 4.9 | -0.14 | 0.66 |  | 0 X |  | 0 X | 0 X |  | 0 X |  |  | 0 X |  | 0 X |
| DPM[26] |  |  | 5.2* | 5.1* | 0.08 | 0.05 | 1 🗸 |  |  | 1 🗸 | 1 🗸 |  |  | 1 🗸 | 1 🗸 |  |  | 1 🗸 |
| DPP[27] |  |  | 5.3* | 5.3* | 0.05 | <0.001 | 0 🗸 |  |  | 0 🗸 | 0 🗸 |  |  | 0 🗸 | 0 🗸 |  |  | 0 🗸 |
| Dutta et al. 2014[28] | 6.1* | 6.2* | 6.1* | 5.8* | 0.41 | 0.02 |  | 1 🗸 |  | 1 🗸 |  | 1 🗸 |  | 1 🗸 |  | 1 🗸 |  | 1 🗸 |
| Hegde et al. 2013[29] | 5.8 | 5.6 | 5.3 | 5.1 | 0.00 | 0.04 |  | 0 🗸 |  | 0 🗸 |  | 0 🗸 |  | 0 🗸 |  | 0 🗸 |  | 0 🗸 |
| Hurst et al. 2010[30] | 4.9 | 5.0 | 4.7 | 4.8 |  | 0.82 |  | X |  | X |  | X |  | X |  | X |  | X |
| IDPP-1 [16] | 5.5 | 6.5 | 5.4 | 6.1 | 0.23 | 0.03 |  | 1 🗸 | 1 🗸 |  | 1 🗸 |  | 1 🗸 |  | 1 🗸 |  | 1 🗸 |  |
| IDPP-2 [36] |  |  | 5.7 | 5.9 | -0.24 | ≥0.05 |  | 0 X | 0 X |  | 0 X |  | 0 X |  | 0 X |  | 0 X |  |
| Islam et al. 2016 [37] | 5.8 | 5.69 | 5.8 | 5.37 | 1.5 | <0.001 |  | 3 🗸 |  | 3 🗸 |  | 3 🗸 |  | 3 🗸 |  | 1 🗸 |  | 1 🗸 |
| InnvaDiab-DE-PLAN[33] | 5.5 | 5.7 | 5.6 | 5.4 | 2.78 | 0.02 |  | 3 🗸 | 3 🗸 |  |  | 3 🗸 |  | 3 🗸 | 3 🗸 |  | 3 🗸 |  |
| PAMH[31] | 5.5 |  | 5.3 |  |  | 0.30 |  | X |  | X |  | X |  | X |  | X |  | X |
| PODOSA[17] | 5.8 | 6.0 | 5.8 | 5.8 | 0.12 | 0.34 | 0 X | 0 X | 0 X |  | 0 X |  | 0 X |  | 0 X |  | 0 X |  |
| RICE[34] | 6.2* | 6.3* | 6.4* | 4.9* | 0.70 | <0.01 | 2 🗸 |  | 2 🗸 |  |  | 2 🗸 | 2 🗸 |  |  | 2 🗸 | 2 🗸 |  |
| **2-h Glucose (mmol/l)** |  |  |  |  |  |  |  |  |  |  |  |  |  |  |  |  |  |  |
| DH!AAN[13] | 5.9 | 6.0 | 6.3 | 6.3 | 0.06 | 0.44 |  | 0 X |  | 0 X | 0 X |  | 0 X |  |  | 0 X |  | 0 X |
| Dutta et al. 2014[28] | 8.6* | 9.3* | 8.5* | 8.0* | 0.57 | <0.001 |  | 2 🗸 |  | 2 🗸 |  | 2 🗸 |  | 2 🗸 |  | 2 🗸 |  | 2 🗸 |
|  |  |  |  |  |  |  |  |  |  |  |  |  |  |  |  |  |  |  |
| Hegde et al. 2013[29] | 6.8 | 6.5 | 7.2 | 6.6 | 0.26 | 0.59 |  | 1 X |  | 1 X |  | 1 X |  | 1 X |  | 1 X |  | 1 X |
| IDPP-1[16] | 8.6 | 10.9 | 8.5 | 9.9 | 0.35 | 0.01 | 1 🗸 |  |  | 1 🗸 | 1 🗸 |  | 1 🗸 |  | 1 🗸 |  | 1 🗸 |  |
| IDPP-2[36] |  |  | 8.9 | 9.5 | -0.68 | >0.05 | 0 X |  | 0 X |  | 0 X |  | 0 X |  | 0 X |  | 0 X |  |
| InnvaDiab-DE-PLAN[33] | 8.4 | 8.2 | 8.5 | 8.0 | 1.15 | 0.19 | 3 X |  | 3 X |  |  | 3 X |  | 3 X | 3 X |  |  | 3 X |
| PAMH[31] | 7.6 |  | 6.4 |  |  | 0.40 |  | X |  | X |  | X |  | X |  | X |  | X |
| PODOSA[17] | 8.3 | 8.1 | 8.2 | 7.4 | 0.26 | 0.14 | 1 X |  | 1 X |  | 1 X |  | 1 X |  | 1 X |  | 1 X |  |
| **Insulin (pmol/l)** |  |  |  |  |  |  |  |  |  |  |  |  |  |  |  |  |  |  |
| DH!AAN[13] | 14 | 13 | 15 | 13 | 0.11 | 0.12 |  | 0 X |  | 0 X | 0 X |  | 0 X |  |  | 0 X |  | 0 X |
| Hurst et al. 2010[30] | 83* | 91* | 92* | 78* |  | 0.02 |  | 🗸 |  | 🗸 |  | 🗸 |  | 🗸 |  | 🗸 |  | 🗸 |
| InnvaDiab-DE-PLAN[33] | 116.3 | 123.3 | 127.6 | 121.8 | 2.08 | 0.04 | 3 🗸 |  | 3 🗸 |  |  | 3 🗸 |  | 3 🗸 | 3 🗸 |  |  | 3 🗸 |
| PAMH[31] | 107 |  | 101 |  |  | 0.50 |  | X |  | X |  | X |  | X |  | X |  | X |
| **HbA1c (%) [mmol/mol]** |  |  |  |  |  |  |  |  |  |  |  |  |  |  |  |  |  |  |
| DH!AAN[13] | 5.7 | 5.7 | 5.7 | 5.7 | 0.00 | 0.99 |  | 0 X |  | 0 X | 0 X |  | 0 X |  |  | 0 X |  | 0 X |
| Dutta et al. 2014[28] | 6.05 | 6.43 | 6.15 | 6.34 | 0.25 | ≥0.05 |  | 1 X |  | 1 X |  | 1 X |  | 1 X |  | 1 X |  | 1 X |
| Hegde et al. 2013[29] | 6.2 | 6.2 | 6.3 | 6.2 | 0.26 | 0.40 |  | 1 X |  | 1 X |  | 1 X |  | 1 X |  | 1 X |  | 1 X |
| IDPP-1[16] | 6.2 | 6.4 | 6.1 | 6.1 | 0.24 | 0.02 |  | 1 🗸 | 1 🗸 |  | 1 🗸 |  | 1 🗸 |  | 1 🗸 |  | 1 🗸 |  |
| IDPP-2[36] |  |  | 5.8 | 6.3 | -0.92 | <0.001 |  | 0 X | 0 X |  | 0 X |  | 0 X |  | 0 X |  | 0 X |  |
| InnvaDiab-DE-PLAN[33] | 5.4 | 5.5 | 5.4 | 5.4 | 1.37 | 0.88 |  | 1 X | 1 X |  |  | 1 X |  | 1 X | 1 X |  |  | 1 X |
| PAMH[31] | 6.7 |  | 5.6 |  |  | 0.09 |  | X |  | X |  | X |  | X |  | X |  | X |
| **HOMA IR** |  |  |  |  |  |  |  |  |  |  |  |  |  |  |  |  |  |  |
| DH!AAN[13] | 3.3 | 2.8 | 3.6 | 2.7 | 0.20 | 0.22 |  | 0 X |  | 0 X | 0 X |  | 0 X |  |  | 0 X |  | 0 X |
| Dutta et al. 2014[28] | 1.47 | 1.15 | 1.46 | 0.96 | 0.26 | 0.01 |  | 1 🗸 |  | 1 🗸 |  | 1 🗸 |  | 1 🗸 |  | 1 🗸 |  | 1 🗸 |
| Hurst et al. 2010[30] | 1.5 | 1.7 | 1.7 | 1.5 |  | 0.02 |  | 🗸 |  | 🗸 |  | 🗸 |  | 🗸 |  | 🗸 |  | 🗸 |
| IDPP-1[16] | 5.4 | 4.6 | 4.8 | 4.5 |  | 0.13 |  | X | X |  | X |  | X |  | X |  | X |  |
| Islam et al. 2016[37] | 1.15 | 1.09 | 1.15 | 1.13 | 0.94 | 0.001 |  | 3 🗸 |  | 3 🗸 |  | 3 🗸 |  | 3 🗸 |  | 3 🗸 |  | 3 🗸 |
| **HOMA 2β** |  |  |  |  |  |  |  |  |  |  |  |  |  |  |  |  |  |  |
| Dutta et al. 2014[28] | 78.3 | 72.25 | 80.2 | 72.1 | 0.06 | ≥0.05 |  | 0 X |  | 0 X |  | 0 X |  | 0 X |  | 0 X |  | 0 X |
| Hurst et al. 2010[30] | 144 | 149 | 163 | 152 |  | 0.09 |  | X |  | X |  | X |  | X |  | X |  | X |
| **HOMA S** |  |  |  |  |  |  |  |  |  |  |  |  |  |  |  |  |  |  |
| Hurst et al. 2010[30] | 65.9 | 60.4 | 60.6 | 68 |  | 0.003 |  | 🗸 |  | 🗸 |  | 🗸 |  | 🗸 |  | 🗸 |  | 🗸 |
| Islam et al. 2016[37] | 86.4 | 88.6 | 86.5 | 90.4 | 1.60 | 0.01 |  | 3🗸 |  | 3🗸 |  | 3🗸 |  | 3🗸 |  | 3🗸 |  | 3🗸 |
| **C-peptide (pmol/l)** |  |  |  |  |  |  |  |  |  |  |  |  |  |  |  |  |  |  |
| Hurst et al. 2010[30] | 830 | 860 | 810 | 810 |  | 0.15 |  | X |  | X |  | X |  | X |  | X |  | X |
| InnvaDiab-DE-PLAN[33] | 839.9 | 914.6 | 855 | 878.5 | 1.44 | 0.29 |  | 3 X |  | 3 X |  | 3 X |  | 3 X | 3 X |  |  | 3 X |
| PAMH[31] | 1017 |  | 993 |  |  | 0.10 |  | X |  | X |  | X |  | X |  | X |  | X |
| **Weight (kg)** |  |  |  |  |  |  |  |  |  |  |  |  |  |  |  |  |  |  |
| DH!AAN[13] | 73.7 | 74.1 | 76.3 | 76.1 | 0.05 | 0.08 |  | 0 X |  | 0 X | 0 X |  | 0 X |  |  | 0 X |  | 0 X |
| IDPP-2[36] |  |  | 68.7 | 68.3 | 0.04 | ≥0.05 |  | 0 X | 0 X |  | 0 X |  | 0 X |  | 0 X |  | 0 X |  |
| PAMH[31] | 84.1 |  | 83.7 |  |  | 0.01 |  | 🗸 |  | 🗸 |  | 🗸 |  | 🗸 |  | 🗸 |  | 🗸 |
| PODOSA[17] | 80.7 | 81.0 | 79.8 | 78.8 | 0.08 | 0.01 | 0 🗸 |  | 0 🗸 |  | 0 🗸 |  | 0 🗸 |  | 0 🗸 |  | 0 🗸 |  |
| RICE[34] | 79.3* | 78.8* | 72.7* | 70.5* | 0.15 | 0.10 | 0 X |  | 0 X |  | 0 X |  |  | 0 X |  | 0 X | 0 X |  |
| **BMI (kg/m2)** |  |  |  |  |  |  |  |  |  |  |  |  |  |  |  |  |  |  |
| DH!AAN[13] | 27.2 | 27.4 | 28.1 | 28 | 0.08 | 0.09 |  | 0 X |  | 0 X | 0 X |  | 0 X |  |  | 0 X |  | 0 X |
| DPM[26] |  |  | 20.6 | 20.8 | -0.05 | 0.004 | 0 X |  |  | 0 X | 0 X |  |  | 0 X | 0 X |  |  | 0 X |
| DPP[27] |  |  | 20.7 | 20.6 | 0.02 | <0.01 | 0 🗸 |  |  | 0 🗸 | 0 🗸 |  |  | 0 🗸 | 0 🗸 |  |  | 0 🗸 |
| Dutta et al. 2014[28] | 26.8 | 26.7 | 26.3 | 25.1 | 0.24 | ≥0.05 |  | 1 X |  | 1 X |  | 1 X |  | 1 X |  | 1 X |  | 1 X |
| Hegde et al. 2013[29] | 26.8 | 26.3 | 27.2 | 26.2 | 0.13 | 0.01 |  | 0 🗸 |  | 0 🗸 |  | 0 🗸 |  | 0 🗸 |  | 0 🗸 |  | 0 🗸 |
| IDPP-1[16] | 26.0 | 26.4 | 25.5 | 25.7 | 0.06 | 0.04 |  | 0 🗸 | 0 🗸 |  | 0 🗸 |  | 0 🗸 |  | 0 🗸 |  | 0 🗸 |  |
| IDPP-2[36] |  |  | 26.2 | 25.9 | 0.03 | ≥0.05 |  |  | 0 X |  | 0 X |  | 0 X |  | 0 X |  | 0 X |  |
| InnvaDiab-DE-PLAN[33] | 29.7 | 29.7 | 29.4 | 29.1 | 0.34 | 0.18 |  | 1 X | 1 X |  |  | 1 X |  | 1 X | 1 X |  |  | 1 X |
| PAMH[31] | 27.4 |  | 27.1 |  |  | <0.01 |  | 🗸 |  | 🗸 |  | 🗸 |  | 🗸 |  | 🗸 |  | 🗸 |
| PODOSA[17] | 30.5 | 30.7 | 30.6 | 30.2 | 0.11 | 0.01 | 0 🗸 |  | 0 🗸 |  | 0 🗸 |  | 0 🗸 |  | 0 🗸 |  | 0 🗸 |  |
| Ramachandran et al. 2013[25] | 25.8 | 25 | 25.8 | 25 | 0.00 |  |  | 0 | 0 |  | 0 |  | 0 |  | 0 |  | 0 |  |
| RICE[34] | 28.6 | 28.5 | 27.8 | 27 | 0.20 | 0.08 | 0 X |  | 0 X |  |  | 0 X | 0 X |  |  | 0 X | 0 X |  |
| **WC (cm)** |  |  |  |  |  |  |  |  |  |  |  |  |  |  |  |  |  |  |
| DH!AAN[13] | 92 | 94 | 94 | 95 | 0.09 | 0.50 |  | 0 X |  | 0 X | 0 X |  | 0 X |  |  | 0 X |  | 0 X |
| DPM[26] |  |  | 76* | 71* | 0.43 | 0.001 | 1 🗸 |  |  | 1 🗸 | 1 🗸 |  |  | 1 🗸 | 1 🗸 |  |  | 1 🗸 |
| DPP[27] |  |  | 75* | 75* | 0.05 | 0.01 | 0 🗸 |  |  | 0 🗸 | 0 🗸 |  |  | 0 🗸 | 0 🗸 |  |  | 0 🗸 |
| Hegde et al. 2013[29] | 89.7 | 90 | 88.9 | 89.4 | -0.02 | 0.03 |  | 0 🗸 |  | 0 🗸 |  | 0 🗸 |  | 0 🗸 |  | 0 🗸 |  | 0 🗸 |
| IDPP-1[16] | 89.3 | 90.8 | 89.1 | 90 | 0.07 | 0.43 |  | 0 X | 0 X |  | 0 X |  | 0 X |  | 0 X |  | 0 X |  |
| IDPP-2[36] |  |  | 91.1 | 90.1 | 0.14 | ≥0.05 |  | 0 X | 0 X |  | 0 X |  | 0 X |  | 0 X |  | 0 X |  |
| InnvaDiab-DE-PLAN[33] | 96.2 | 96.8 | 95.3 | 95.1 | 0.62 | 0.18 |  | 1 X | 1 X |  |  | 1 X |  | 1 X | 1 X |  |  | 1 X |
| PAMH[31] | 99 |  | 98 |  |  | 0.01 |  | 🗸 |  | 🗸 |  | 🗸 |  | 🗸 |  | 🗸 |  | 🗸 |
| PODOSA[17] | 103.3 | 102.9 | 102.7 | 100.5 | 0.16 | 0.01 | 0 🗸 |  | 0 🗸 |  | 0 🗸 |  | 0 🗸 |  | 0 🗸 |  | 0 🗸 |  |
| Ramachandran et al. 2013[25] | 92.7 | 92.6 | 92.6 | 92.6 | -0.01 |  |  | 0 | 0 |  | 0 |  | 0 |  | 0 |  | 0 |  |
| RICE[34] | 93* | 90* | 93* | 88* | 0.19 | 0.39 | 0 X |  | 0 X |  |  | 0 X | 0 X |  |  | 0 X | 0 X |  |
| **HC (cm)** |  |  |  |  |  |  |  |  |  |  |  |  |  |  |  |  |  |  |
| DH!AAN[13] | 98 | 98 | 99 | 99 | 0.00 | 0.13 |  | 0 X |  | 0 X | 0 X |  | 0 X |  |  | 0 X |  | 0 X |
| DPM[26] |  |  | 88* | 85* | 0.37 | 0.001 | 1 🗸 |  |  | 1 🗸 | 1 🗸 |  |  | 1 🗸 | 1 🗸 |  |  | 1 🗸 |
| **TC (cm)** |  |  |  |  |  |  |  |  |  |  |  |  |  |  |  |  |  |  |
| DPM[26] |  |  | 45* | 42* | 0.42 | 0.001 | 1 🗸 |  |  | 1 🗸 | 1 🗸 |  |  | 1 🗸 | 1 🗸 |  |  | 1 🗸 |
| PODOSA[17] | 107.3 | 106.7 | 106.9 | 104.5 | 0.18 | 0.01 | 0 🗸 |  | 0 🗸 |  | 0 🗸 |  | 0 🗸 |  | 0 🗸 |  | 0 🗸 |  |
| **WHR** |  |  |  |  |  |  |  |  |  |  |  |  |  |  |  |  |  |  |
| DPM[26] |  |  | 0.86 | 0.83 | 0.38 | 0.001 | 1 🗸 |  |  | 1 🗸 | 1 🗸 |  |  | 1 🗸 | 1 🗸 |  |  | 1 🗸 |
| Dutta et al. 2014[28] | 0.91 |  | 0.89 |  |  | ≥0.05 |  | X |  | X |  | X |  | X |  | X |  | X |
| Hegde et al. 2013[29] | 0.92 | 0.93 | 0.89 | 0.88 | 0.32 | 0.08 |  | 1 X |  | 1 X |  | 1 X |  | 1 X |  | 1 X |  | 1 X |
| PODOSA[17] | 0.96 | 0.96 | 0.96 | 0.96 | 0.00 | 0.68 | 0 X |  | 0 X |  | 0 X |  | 0 X |  | 0 X |  | 0 X |  |
| **BF (%)** |  |  |  |  |  |  |  |  |  |  |  |  |  |  |  |  |  |  |
| DH!AAN[13] | 36 | 35.1 | 36.6 | 35.7 | 0.00 | 0.84 |  | 0 X |  | 0 X | 0 X |  | 0 X |  |  | 0 X |  | 0 X |
| IDPP-2[36] men |  |  | 31.2 | 31.2 | 0.00 | ≥0.05 |  | 0 X | 0 X |  | 0 X |  | 0 X |  | 0 X |  | 0 X |  |
| women |  |  | 45.9 | 44.7 | 0.16 | ≥0.05 |  |  |  |  |  |  |  |  |  |  |  |  |

0, 1, 2 or 3 are shown to indicate the magnitude of the effect size, which corresponds to none (<0.2), small (>0.2), medium (>0.5) or large (0.8) A 🗸 is shown to indicate that the study reported a significant improved p-value and a X is shown to indicate that there was no significant improved p-value. Yes and no were used to indicate whether the component was included in the study. Studies on vitamin D and physical activity were included in the evaluation of effects of components but did not include any of the evaluated components**.** T2D, type 2 diabetes; 2-h glucose, 2-h post 75g glucose blood glucose; IGT, Impaired Glucose Tolerance; IFG, Impaired Fasting Glucose; BMI, Body Mass Index; WC, Waist Circumference; HC, Hip Circumference; TC, Thigh Circumference; WHR, Waist Hip Ratio; BF, Body Fat; Out, Outcome; I, Intervention group; C, Control group; Base, Baseline; ES, Effect size *Original units were converted to SI units.
