# Supplementary material for: Dietary and physical activity recommendations to prevent type 2 diabetes in South Asian adults: A systematic review
Source: PLoS One. 2018 Jul 16;13(7):e0200681. doi: 10.1371/journal.pone.0200681 (PMC6047810; doi:10.1371/journal.pone.0200681)
Supplement: S3 Table — Data are the number of studies reporting the specific component. Components were included in the table, if these were recommended by more than one study. (DOC) [file pone.0200681.s006.doc]

**S3 Table. Patterns according to included components.**

|  | **Migrant status** | | **Geographical setting** | | | | **Ethnicity** | |
| --- | --- | --- | --- | --- | --- | --- | --- | --- |
|  | **Migrant (6)** | **Non-migrant (8)** | **Europe (4)** | **U.S. (1)** | **Asia (8)** | **Oceania (1)** | **Indian (9)** | **Other (5)** |
| Protein | 2 | 3 | 1 | 1 | 3 | 0 | 4 | 1 |
| Fat | 5 | 5 | 3 | 2 | 5 | 0 | 7 | 3 |
| Carbohydrates | 2 | 3 | 2 | 0 | 3 | 0 | 3 | 2 |
| Sugar | 3 | 5 | 2 | 1 | 5 | 0 | 6 | 2 |
| Salt | 2 | 0 | 1 | 1 | 0 | 0 | 1 | 1 |
| Vitamins | 2 | 1 | 1 | 0 | 1 | 1 | 0 | 2 |
| Fibre | 2 | 5 | 2 | 0 | 5 | 0 | 5 | 2 |
| Fruits | 4 | 3 | 2 | 2 | 3 | 0 | 5 | 2 |
| Vegetables | 5 | 5 | 3 | 2 | 5 | 0 | 7 | 3 |
| Whole grains | 3 | 5 | 2 | 1 | 5 | 0 | 4 | 4 |
| Cereal and grains | 0 | 3 | 0 | 0 | 3 | 0 | 3 | 0 |
| Legumes | 2 | 5 | 2 | 0 | 5 | 0 | 5 | 2 |
| Portion sizes | 2 | 2 | 1 | 1 | 2 | 0 | 3 | 1 |
| Balanced | 2 | 3 | 1 | 1 | 3 | 0 | 4 | 1 |

Data are the number of studies reporting the specific component. Components were included in the table, if these were recommended by more than one study.
