# Supplementary material for: Dietary and physical activity recommendations to prevent type 2 diabetes in South Asian adults: A systematic review
Source: PLoS One. 2018 Jul 16;13(7):e0200681. doi: 10.1371/journal.pone.0200681 (PMC6047810; doi:10.1371/journal.pone.0200681)
Supplement: S4 Table — (DOC) [file pone.0200681.s007.doc]

**S4 Table. Recommended components NICE guideline [8].**

| **NICE Guideline Recommended Components** |
| --- |
| **Dietary guidelines**   - Base meals on starchy foods such as potatoes, bread, rice and pasta, choosing wholegrain where possible. - Eat fibre-rich foods such as oats, beans, peas, lentils, grains, seeds, fruit, vegetables, wholegrain bread and brown rice and pasta. - Eat at least five portions of a variety of fruit and vegetables each day, in place of foods higher in fat and calories. - Adopt a low-fat diet. - Avoid increasing fat or calorie intake. - Consume as little as possible fried food; drinks and confectionery high in added sugars (such as cakes, pastries and sugar-sweetened drinks); and other food high in fat and sugar (such as some take-away and fast foods). - Minimise calorie intake from alcohol. - Watch the portion size of meals and snacks, and how often they are eating throughout the day. - Eat breakfast.   **Physical activity guidelines**   - Make activities they enjoy, such as walking, cycling, swimming, aerobics and gardening, a routine part of life and build other activity into their daily routine – for example by taking the stairs instead of the lift or taking a walk at lunchtime. - Minimise sedentary activities, such as sitting for long periods watching television, at a computer or playing video games. - Use physically active forms of travel such as walking and cycling. - (National recommendations) Accumulate at least 30 minutes of at least moderate-intensity physical activity on 5 or more days of the week. |
|  |
